# Synthesis, Physical Properties, and Reactivity of Stable, π-Conjugated, Carbon-Centered Radicals

**DOI:** 10.3390/molecules24040665

**Published:** 2019-02-13

**Authors:** Takashi Kubo

**Affiliations:** Department of Chemistry, Graduate School of Science, Osaka University, Toyonaka, Osaka 560-0043, Japan; kubo@chem.sci.osaka-u.ac.jp

**Keywords:** π-conjugated radicals, hydrocarbon radicals, persistent, anthryl, phenalenyl, fluorenyl

## Abstract

Recently, long-lived, organic radical species have attracted much attention from chemists and material scientists because of their unique electronic properties derived from their magnetic spin and singly occupied molecular orbitals. Most stable and persistent organic radicals are heteroatom-centered radicals, whereas carbon-centered radicals are generally very reactive and therefore have had limited applications. Because the physical properties of carbon-centered radicals depend predominantly on the topology of the π-electron array, the development of new carbon-centered radicals is key to new basic molecular skeletons that promise novel and diverse applications of spin materials. This account summarizes our recent studies on the development of novel carbon-centered radicals, including phenalenyl, fluorenyl, and triarylmethyl radicals.

## 1. Introduction

Organic radical species are generally recognized as highly reactive, intermediate species. However, recently, functional materials taking advantage of the feature of open-shell electronic structure have attracted much attention from chemists and material scientists; therefore, the development of novel, long-lived, organic, radical species becomes more important [1,2,3,4,5,6,7]. Nitronyl nitroxides, galvinoxyl, and DPPH are well known as stable, organic, radical species, which are commercially available chemicals. These stable radical species are “heteroatom-centered radicals”, in which unpaired electrons are mainly distributed on heteroatoms. On the other hand, “carbon-centered radicals”, in which unpaired electrons exist only on carbon atoms, are highly reactive and difficult to handle and therefore have had limited applications. However, because the physical properties of carbon-centered radicals depend predominantly on the topology of the π-electron array, the development of new carbon-centered radicals is key to new basic molecular skeletons that promise novel and diverse applications of spin materials. By investigating the physical properties and reactivity of newly developed radical species, it becomes possible to find new applications, leading to the creation of new functional materials.

There are two definitions of stability of radicals: thermodynamic stability and kinetic stability. Thermodynamic stability is related to the degree of generation of radical species; for instance, it determines how many monomers are formed when the radical dimer is cleaved into a monomer. On the other hand, kinetic stability is related to the lifetime of radical species. The thermodynamic stability of radical species can be evaluated by the indices such as bond dissociation energy (BDE) and radical stabilization energy (RSE) [8]. The BDE is the energy required for homolytic cleavage of R–H into R^•^ and H^•^, whereas the RSE is expressed by the difference between the BDE of a radical species of interest and the BDE of the methyl radical. Either BDEs or RSEs may be used for evaluating the thermodynamic stability of radical species. However, RSE might be easier to evaluate the stability of radicals because RSEs indicate the relative stability to the methyl radical. Table 1 shows RSE values of selected carbon-centered radicals calculated at the second-order, restricted, open-shell, Møller–Plesset (ROMP2) level of theory. Generally, the larger the delocalization of unpaired electrons, the more thermodynamically stabilized the radical species, and the RSE shows a larger negative value.

Research on stable organic radicals started with the triphenylmethyl radical (**1**), which was discovered by Gomberg in 1900 [9]. Although **1** reacts promptly with molecular oxygen, the radical exists for a long period of time under degassed conditions or in an inert gas atmosphere. In solution, **1** is in equilibrium with a dimer and dissociates mostly into a monomeric radical in dilute solution (about 10^−^^5^ M), indicating high thermodynamic stability of **1**. The RSE value of **1** is calculated to be −103.4 kJ mol^−^^1^.

## 2. Phenalenyl Radical

The phenalenyl radical (**2**) is a spin-delocalized hydrocarbon radical with *D*_3h_ symmetry. Due to its relatively long lifespan, **2** has attracted attention from organic chemists in terms of its chemical reactivity of radical species [10]. On the other hand, in 1973, Haddon proposed that a one-dimensional (1D) stack of organic radicals should show metallic behavior and also showed that **2** is a good model compound for his theoretical prediction [11]. After that, many researchers became interested in the physical properties of **1** [12,13,14,15,16]. However, organic radicals generally dimerize to form closed-shell compounds. Therefore, a considerable effort is required for the molecular design to obtain a one-dimensional chain of radicals. This section describes our recent studies on self-association behaviors of phenalenyl radicals, aiming at constructing a one-dimensional chain as a final goal.

### 2.1. Electronic Structure of Phenalenyl Radical

**Figure 1:**
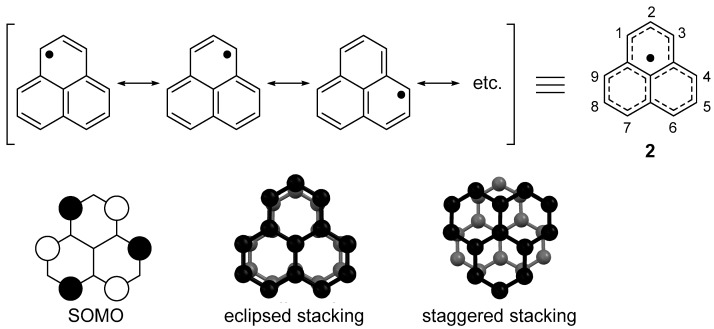
Resonance structure and singly occupied molecular orbital (SOMO) of phenalenyl radical (**2**).

The phenalenyl radical (**2**) is an odd-alternant hydrocarbon radical, and owing to its highly symmetric structure, an unpaired electron is delocalized on six equivalent carbon atoms (1, 3, 4, 6, 7, and 9-positions: the α-position), as shown in Figure 1. This spin delocalization leads to the high thermodynamic stability of **2**, and indeed, the RSE value of **2** is −201.6 kJ mol^−1^, which is almost twice that of **1**. The Hückel molecular orbital (HMO) calculation shows a singly occupied molecular orbital (SOMO) with an energy of α + 0β. The SOMO of **2** is distributed only on the α-position, and the MO coefficients of the other atoms are zero, consistent with the resonance form shown in Figure 1. The feature of the SOMO of **2** is a hexagonal arrangement, which allows perfect orbital overlap in both eclipsed and staggered stacking motifs. The staggered stacking is more favorable to short π–π contact (face-to-face contact) than the eclipsed stacking due to smaller atom–atom repulsion.

### 2.2. Dimerizaion Behavior of Phenalenyl Radicals

**Figure 2:**
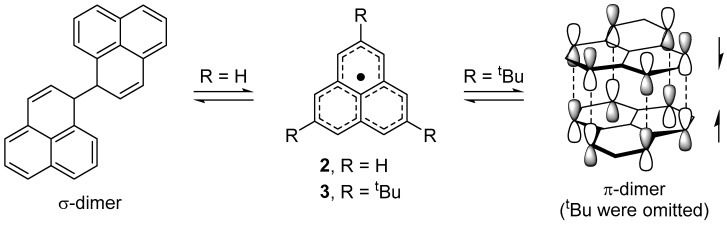
Dimerization mode of phenalenyl radicals.

The parent phenalenyl radical **2** is a long-lived species in the absence of air and is in equilibrium with a σ-bonded dimer (σ-dimer) in solution state (Figure 2) [17]. The thermodynamically stabilized nature of **2** suggests that the structural modification, such as the introduction of substituent groups, may lead to another association mode other than a σ-dimer form. In fact, the introduction of bulky *tert*-butyl groups at 2,5,8-positions afforded a face-to-face π-dimer in the solid [18] and solution states [19] (Figure 2). Two phenalenyl planes are superimposed at the separation distance of 3.25 Å in the staggered stacking manner. The π-dimer of the *tert*-butyl derivative (**3**) shows a deep blue color and an intense absorption band is observed at 612 nm in the solid state. A quantum chemical calculation (ZINDO/S) for the π-dimer predicted a fully allowed absorption band around 600 nm, with the transition moment along the direction connecting the centers of the phenalenyl rings. The intense band can be assigned to an electronic transition from HOMO to LUMO, which are newly formed by the orbital interaction of a pair of SOMO in the π-dimer. The short π–π contact and the orbital splitting indicate the adequate covalent bonding interaction of two unpaired electrons [20,21].

**Figure 3:**
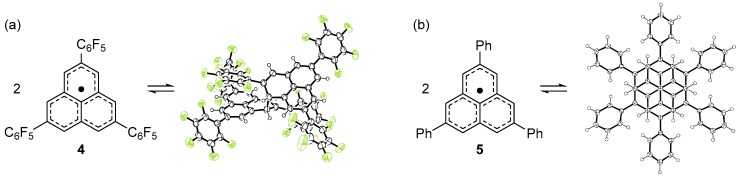
Dimerization mode of (**a**) **4** and (**b**) **5**.

Because a π-dimer is the smallest unit of a 1D stack, we expected that a chemical modification such as the introduction of substituent groups could lead to an equidistantly stacked 1D chain [22]. As a first trial, we decided to introduce a pentafluorophenyl (C_6_F_5_) group at 2,5,8-positions [23]. Highly polarized C_6_F_5_ group would afford aggregates exceeding a π-dimer due to the strong electrostatic interaction between the C_6_F_5_ groups. Indeed, C_6_F_5_ groups serve as a supramolecular synthon, giving rise to a 1D stack of molecules [24,25]. The key precursor of the target radical (**4**) was synthesized from 2,7-dibromonaphthalene in nine steps. The dehydrogenation of the precursor with dichlorodicyano-*p*-benzoquinone (DDQ) afforded pale yellow crystals, which is in sharp contrast to the blue color of the π-dimer of **3**. The X-ray crystallographic analysis of the yellow crystal showed that **4** adopts a σ-dimer form (**4**_2_) in the solid state (Figure 3a). The ^1^H NMR spectrum of the pale yellow crystals in CDCl_3_ showed six sharp singlet signals in the temperature range 233–298 K, consistent with the σ-dimer form. Thus, **4** prefers a σ-dimer form over a π-dimer form in the solution and solid states. The σ-dimer features a long σ-bond of 1.636(7) Å connecting the two phenalenyl rings, indicating the weakness of the σ-bond. Indeed, a toluene solution of the σ-dimer at room temperature showed a well-resolved multiline ESR spectrum originating from monomeric **4**. The enthalpy and entropy changes for the σ-dimerization of **4** were determined to be −64.0 kJ mol^−1^ and −135 J K^−1^ mol^−1^, respectively, from the temperature dependency of the ESR signal intensity.

We also prepared a phenyl derivative (**5**) of **2** [26]. The radical precursor was prepared from 2,7-diphenylnaphthalene in a similar manner as **4**. The dehydrogenation of the precursor with *p*-chloranil afforded deep blue crystals, contrary to our expectation. The X-ray crystallographic analysis of the blue crystal showed that **5** forms a discreet face-to-face π-dimer (**5**_2_) with a C(α)–C(α) separation of 3.067 Å at 300 K (Figure 3b), much shorter than that of **3**_2_. To elucidate the structure of the dimeric species in the solution state, the variable-temperature ^1^H NMR spectra of **5** was measured in CD_2_Cl_2_. No signal was observed at 273 K for the solution of **5**, whereas at 203 K, two broad signals were observed at δ 7.5 ppm (phenyl protons) and 6.8 ppm (phenalenyl protons). Considering that a similar behavior was observed for the π-dimerization of **3**, the phenyl derivative **5** prefers a π-dimer form over a σ-dimer form in the solution state. The dissolution of **5_2_** in toluene showed a well-resolved multiline ESR spectrum corresponding to monomeric **5** at room temperature. The intensity of the ESR signals of **5** decreased with decreasing temperature and almost disappeared at 170 K due to the formation of a diamagnetic π-dimer. The enthalpy and entropy changes for the dimerization were determined to be −40 kJ mol^−1^ and −75 J K^−1^ mol^−1^, respectively, by the variable-temperature ESR measurements.

**Figure 4:**
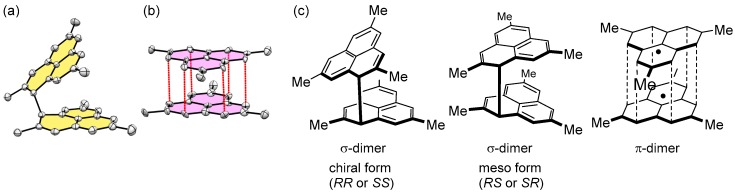
Various dimer forms of **6**. ORTEP (Oak Ridge Thermal-Ellipsoid Plot Program) drawing of (**a**) **6**_2_-σ-chiral and (**b**) **6**_2_-π. (**c**) Three structural isomers of the dimer of **6**.

The steric size of the phenyl group is smaller than that of the C_6_F_5_ group. The preference of π-dimerization for the phenalenyl radical with smaller substituents indicates that the dimerization mode of phenalenyl radicals is not simply determined by a steric factor. Therefore, we decided to prepare a phenalenyl radical derivative with small substituent groups, 2,5,8-trimethyl phenalenyl radical (**6**), in order to investigate the intrinsic dimerization nature of the phenalenyl radical [26,27]. The precursor of **6** was prepared from 2,7-dibromonaphthalene in ten steps. The dehydrogenation reaction of the precursor with *p*-chloranil produced a pink-violet solution, indicating the generation of a π-dimer. However, the concentration of the violet solution yielded colorless platelet crystals, which turned out to be a σ-dimer form by the X-ray crystallographic analysis (Figure 4a). When the colorless plates were heated at 573 K in a sealed, degassed tube, melting occurred, accompanied by a color change from colorless to purple. Subsequent cooling of the purple liquid afforded purple platelet crystals together with colorless plates. The X-ray crystallographic analysis of the purple plate at 100 K showed that the purple, crystalline state adopts a π-dimer form (Figure 4b) with a short contact of α-carbons (av. 3.054 Å). Thus, **6** can exist in both σ- and π-dimer forms in the solid state. The 1D ^1^H NMR spectrum of **6** recorded in degassed THF-*d*_8_ at 173 K showed a relatively complicated spectral pattern. The spectrum consisted of three sets of signals derived from three dimeric isomers (**6**_2_-σ-chiral, **6**_2_-σ-meso, and **6**_2_-π, shown in Figure 4c), which were unambiguously characterized by the 2D-NMR analyses. From the integration ratio of the 1D ^1^H NMR signals, the most stable isomer was found to be the σ-dimer chiral form (**6**_2_-σ-chiral), and the other two (**6**_2_-σ-meso and **6**_2_-π) were metastable forms. However, the energy differences, estimated from the ratio of the NMR signal intensities and Boltzmann distribution, between the metastable forms relative to the most stable 6_2_-σ-chiral were very small: 0.33 kJ mol^−1^ for **6**_2_-σ-meso and 2.8 kJ mol^−1^ for **6**_2_-π.

**Figure 5:**
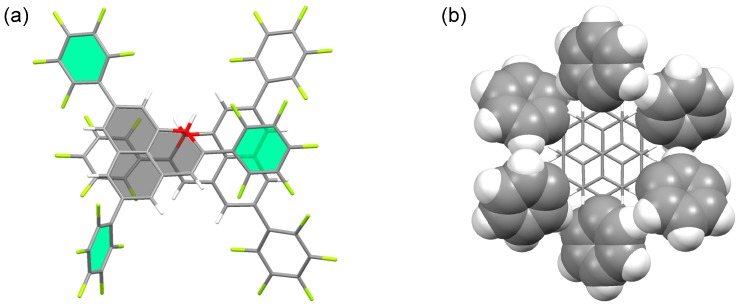
Attractive interactions between radical units of (**a**) **4**_2_ and (**b**) **5**_2_.

From the results of the dimerization behavior of **6**, we concluded that the σ-dimer and π-dimer of the phenalenyl radical are close in energy, and that its dimerization mode can be changed with a slight perturbation of substituents either by a steric or electronic factor. For instance, in **4**_2_, the attractive interaction between the C_6_F_5_ group and naphthalene ring makes the σ-dimer form preferable in energy due to the electrostatic interaction (perfluoroarene–arene interaction), as shown in Figure 5a. On the other hand, **5** adopts the π-dimer form due to a cumulative CH–π interaction among the phenyl substituents (Figure 5b). The M05-2X/6-31G** calculation showed that the total stabilization energies by the substituents in **4**_2_ and **5**_2_ are 35 kJ mol^−1^ and 43 kJ mol^−1^, respectively.

### 2.3. One-Dimensional Stack of Phenalenyl Radicals

**Figure 6:**
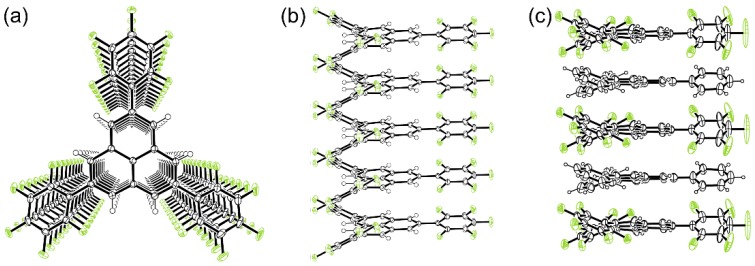
One-dimensional stack of **4**, (**a**) Top view and (**b**) side view. (**c**) Side view of alternating one-dimensional stack of **4** and **5**.

As mentioned above, phenalenyl radicals can adopt σ-dimer and π-dimer forms. Therefore, we attempted to transform the σ-dimer form of **4** into a π-dimer form by external stimulus [23]. The white powder of **4**_2_ was placed in a sealed, degassed tube and heated at 573 K, resulting in the melting of the power and a color change from white to purple. Subsequent cooling of the resulting purple liquid afforded dark purple needles suitable for the X-ray crystallographic analysis. Surprisingly, **4** was found to be equidistantly stacked with the interplanar distance of 3.503 Å to form a 1D chain (Figure 6a,b). No Peierls transition (that is, dimerization in the 1D stack) was observed even at 10 K. Each 1D chain was surrounded by six adjacent chains, and the 1D chains stick together by a weak C–H•••F–C hydrogen-bonding interaction. Considering that an unpaired electron resides only on the phenalenyl moiety, the C_6_F_5_ groups serve as spacers to separate neighboring radical moieties; thus, an ideal 1D chain of spin-1/2 was achieved. The reason for the regular stack of **4** is the close stacking of polarized C_6_F_5_ groups; the interplanar distance between the C_6_F_5_ groups is 3.252 Å on average. Thus, the C_6_F_5_ substituent groups dominantly control the structure of the 1D chain. Because of the large interplanar distance (3.503 Å) of the phenalenyl radical site, the overlapping of the wave functions between the radicals was small. The band width calculated by the extended HMO method was as small as about 0.29 eV. As a result, the 1D chain of **4** was an insulator.

Pentafluorophenyl and phenyl groups alternately stack in the solid state and are used as supramolecular synthons [24,25]. We heated the mixed powder of **4**_2_ and **5**_2_ at 573 K in a sealed, degassed tube, and subsequent cooling gave rise to brown needles. The X-ray crystallographic analysis of the brown needle at 200 K showed that **4** and **5** stack alternately to form a 1D chain with an interplanar distance of 3.69 Å by virtue of the attractive interaction between C_6_F_5_ and phenyl groups (Figure 6c).

## 3. Fluorenyl Radical

**Figure 7:**
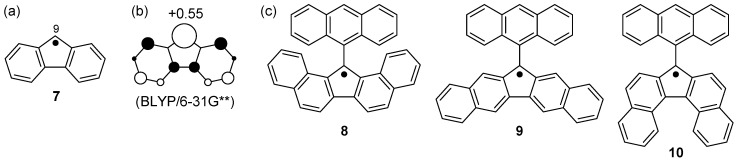
(**a**) Fluorenyl radical (**7**). (**b**) Spin density of **7** calculated by a BLYP/6-31G** method. (**c**) Anthryl-protected dibenzo-fluorenyl radicals (**8**, **9**, **10**).

Fluorenyl radical (**7**), which possesses the same sp^2^ carbon number as the phenalenyl radical (**2**), is also a spin-delocalized hydrocarbon radical. However, unlike **2**, about half of the spin density resides on the 9-position (Figure 7). Therefore, **7** dimerizes promptly, and its σ-dimer does not dissociate into a monomeric radical even in solution [28,29]. Because the RSE of **7** is −93 kJ mol^−1^, the thermodynamic stability of **7** is much lower than that of **2**.

### 3.1. π-Extended Fluorenyl Radicals

To stabilize **7**, which shows remarkable reactivity, a considerable device must be made in the molecular design. It would be possible to prolong the lifetime of **7** by sterically protecting only the spin-localized part by taking advantage of the situation where an unpaired electron is mainly localized on the carbon atom at the 9-position. We decided to introduce an anthryl group at the 9-position, and to expand the π-skeleton to ensure extraordinary stability in air. In addition, three types of dibenzo-fluorenyl radicals (**8**, **9**, **10**), which possess an anthryl substituent group at the central five-membered ring, were prepared by short step syntheses [30]. Interestingly, the difference in the ring-condensation position caused a big difference in lifespan, dimerization mode, and electronic properties of the radicals.

**Figure 8:**
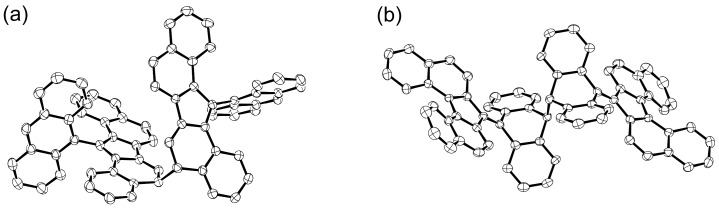
σ-Dimer structures of (**a**) **8** and (**b**) **10** determined by X-ray crystallographic analysis.

**Figure 9:**
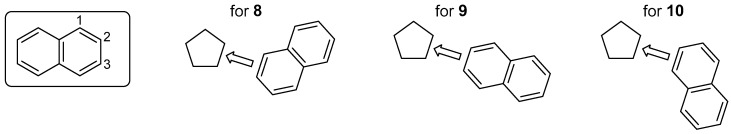
Ring condensation modes for **8**, **9**, and **10**.

**Figure 10:**
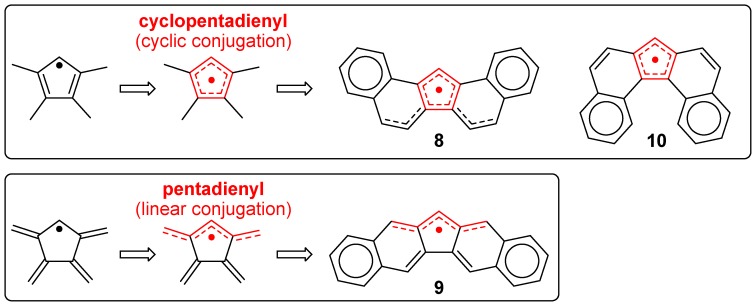
Manner of the delocalization of an unpaired electron based on the spin density calculation (UBLYP/6-31G**//UB3LYP/6-31G**).

The radicals **8**, **9**, and **10** showed relatively long half-life times of 7, 3.5, and 43 days, respectively, in air in the solution state. As for dimerization, **8** formed a σ-bond on the fluorenyl ring, whereas **10** formed a σ-bond on a bent anthracene ring (Figure 8). In contrast, no dimerization reaction was observed in **9**. Regarding physical properties such as redox potentials and UV-Vis-NIR absorptions, **8** and **10** showed similar properties, whereas **9** showed behavior different from those radicals. For instance, the redox waves of **8** and **10** in the cyclic voltammogram appeared on the higher potential side (that is, not easily oxidized and easily reduced) compared with **9**. Regarding the UV-Vis-NIR absorption spectra, **8** and **10** showed weak absorptions in the NIR region, but **9** did not absorb light beyond the visible region. These differences arise from the difference in the manner of condensation of the naphthalene ring with respect to the five-membered ring (Figure 9). It is well-known that naphthalene possesses a larger double-bond character at the 1,2-bond than at the 2,3-bond. Therefore, the five-membered ring condensation to the 1,2 or 2,3 region of naphthalene leads to endo- or exo-double bonds at the five-membered ring, respectively. This difference in the condensation affects the manner of spin delocalization, as shown in Figure 10. Thus, **8** and **10** behave like a cyclopentadienyl radical, whereas **9** behaves like a linear pentadienyl radical. The higher redox potentials of **8** and **10** are attributable to the antiaromatic character of the cyclopentadienyl cation in the cation state and the aromatic character of the cyclopentadienyl anion in the anion state.

**Figure 11:**
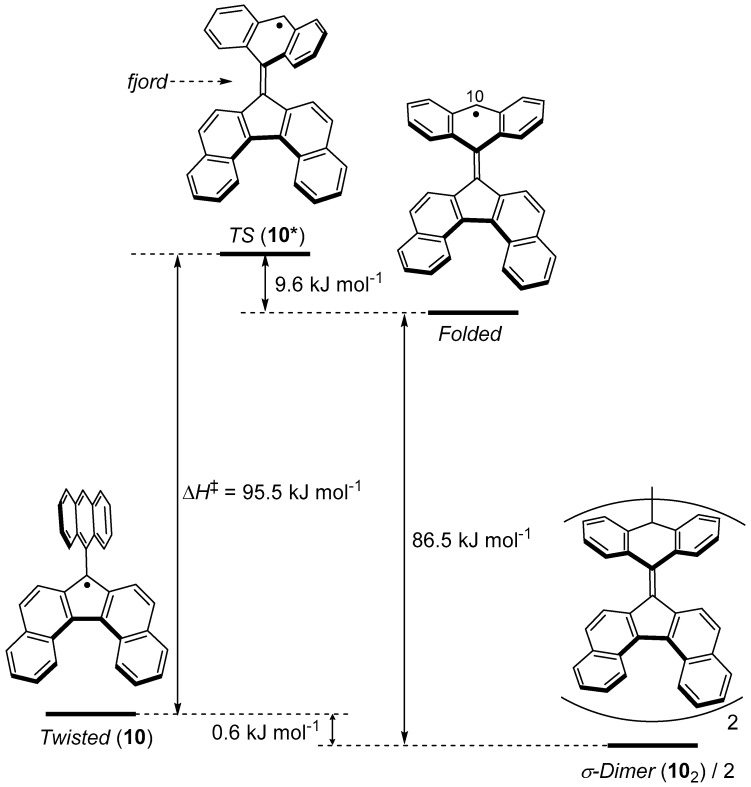
Energy diagram for twisted **10** to **10**_2_ (σ-dimer) calculated with the UB3LYP/6-31G** method.

As shown in Figure 8b, σ-dimerization of **10** occurs not on the dibenzofluorenyl moiety, where an unpaired electron predominantly distributes, but on the anthryl group that is introduced as a sterically protecting group. This σ-dimerization is accompanied by a large structural change of the anthryl group from the planar form to the butterfly form. We estimated the energy profile for the structural change upon the σ-dimerization of **10** by quantum chemical calculations (Figure 11). Although the most stable form of the monomeric **10** is the twisted form, **10** can adopt a metastable folded form, where an unpaired electron resides mostly on the 10-positon of the anthryl group. The structure of the transition state (10*) for the most stable twisted form ⇄ the metastable folded form is close to the folded form, except that the anthryl group is contorted. The calculated enthalpy of activation (Δ*H*^‡^) for the twisted form to 10* was 95.5 kJ mol^−1^ (Figure 11). Although a σ-dimer (**10**_2_) of **10** is more stable than the twisted form, the energy difference is just 0.6 kJ mol^−1^. The low barrier height for the dissociation of **10**_2_ into the monomeric **10** is in line with the fact that the dissolution of the solid of **10**_2_ in a solvent gradually resulted in the equilibrium with the monomeric **10**. The facile dissociation of **10**_2_ suggests the weakness of the σ-bond connecting two anthryl groups. In reality, the solid of **10**_2_ showed mechanochromic behavior. Hard grinding of the crystals of **10**_2_ led to a drastic color change from orange to purple. The purple color corresponds to the color of the monomeric **10**.

### 3.2. Ring Expansion of Fluorenyl Radical

**Figure 12:**
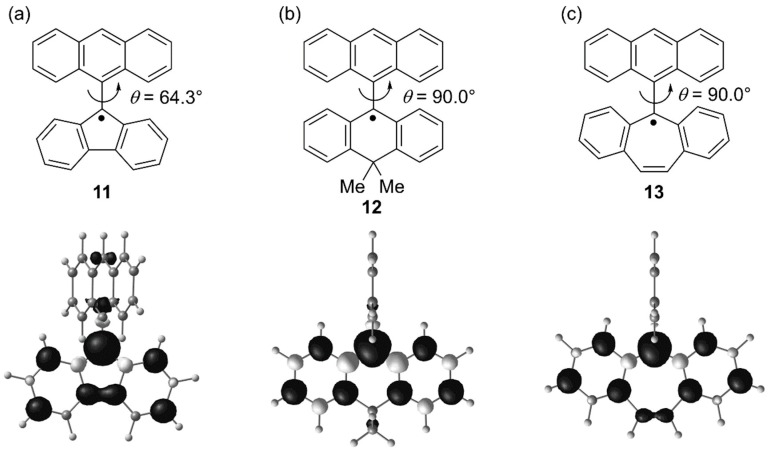
Anthryl-substituted tricyclic radicals (**11** (**a**), **12** (**b**), and **13** (**c**)). Spin density maps calculated by UBLYP/6-31G**//UωB97X-D/6-31G** are also shown.

We also investigated the effect of ring size of the fluorenyl radical on chemical and physical properties. Three kinds of anthracene-attached tricyclic radicals (**11**, **12**, and **13**) were designed and synthesized (Figure 12) [31]. It is well-known that five- and seven-membered rings have electron-deficient and electron-rich properties, respectively. Additionally, the size of the central polygon in the tricyclic system affects steric congestion in the fjord region. These electronic and steric factors would cause the difference in the properties among the tricyclic radicals.

From the decay of the ESR signal intensities in air-saturated toluene, the half-life time of **11** at room temperature was found to be 5.6 days. Considering that the 9-phenylfluorenyl radical rapidly decomposes [32], it seems that the effect of steric protection by an anthryl group on the stability is unexpectedly large. **12** showed a longer half-life time (9 days) than **11** presumably due to the perpendicular conformation between the anthryl group and the tricyclic scaffold [33]. In contrast, **13**, which also adopts the perpendicular conformation, was a short-lived species with a half-life time of about 5 min and was easily oxidized. This high reactivity toward oxygen would be related to the high SOMO level originating from the electron-rich character of the seven-membered ring. The oxidation and reduction potentials (*E*_ox_ and *E*_red_, respectively) determined by cyclic voltammetry are summarized in Table 2. **13** is most easily oxidized among the three radicals, whereas **11** shows the highest electron-accepting ability.

**Figure 13:**
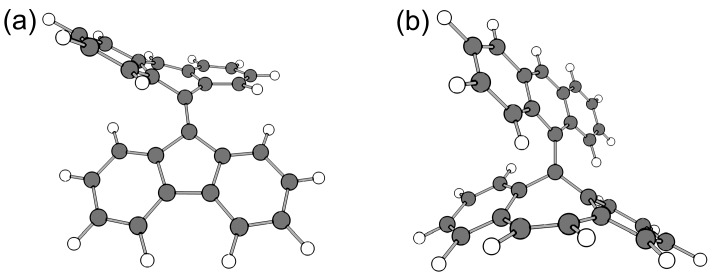
Structures of the transition state of (**a**) **11** and (**b**) **13** calculated at the UBLYP-D3/6-31G** level of theory.

Similar to **10**, **11** also gradually gave rise to a colorless σ-dimer at room temperature in the solution state. In contrast, **12** showed no dimerization behavior for a long period of time. The Δ*H*^‡^ of **12** to a σ-dimer was calculated to be 138 kJ mol^−1^, which is much larger than that of **11** (90 kJ mol^−1^). Therefore, **12** could not exceed the transition state at room temperature. As the central polygon of the tricyclic scaffold becomes a six-membered ring, the steric hindrance of the fjord region became larger than **11**, leading to a higher activation barrier. However, **13**, where the fjord region was anticipated to be crowded more than **11** and **12**, possessed a Δ*H*^‡^ of only 83 kJ mol^−1^. The reason for the unexpectedly low barrier is that in the transition state, the seven-membered ring moiety adopts a tub-like structure, whereby the tricyclic radical moiety greatly bends and the steric hindrance of the fjord region is alleviated (Figure 13). The flexibility of the seven-membered ring moiety is also related to the stability of the σ-dimer. The energy differences between the monomeric radical and the σ-dimer are 64 kJ mol^−1^ and 109 kJ mol^−1^ for **11** and **13**, respectively (the σ-dimers are lower in energy than the monomeric species). Indeed, **13** afforded a σ-dimer quickly at room temperature in the solution state under the degassed condition; furthermore, no dissociation reaction was observed for the σ-dimer.

## 4. Trianthrylmethyl Radical

**Figure 14:**
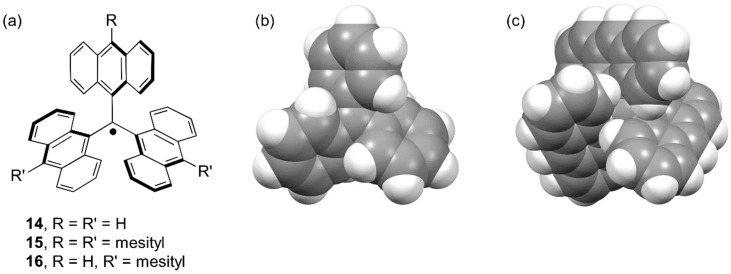
(**a**) Trianthrylmethyl radicals (**14**, **15**, and **16**). Space-filling models of (**b**) **1** and (**c**) **14**.

The stability of the triphenylmethyl radical (**1**) is largely dependent on the steric factor of the phenyl groups [34]. We decided to investigate the effect of larger aryl groups on the stability of the methyl radical and designed a new highly congested hydrocarbon radical, trianthrylmethyl radical (**14**) [35]. As shown in Figure 14, the central carbon atom of **14**, which possesses the largest spin density, is more effectively protected by three anthryl groups than that of **1**. We expected that **14** could be isolated in the solid state with no substituent groups on the anthryl group.

**Figure 15:**
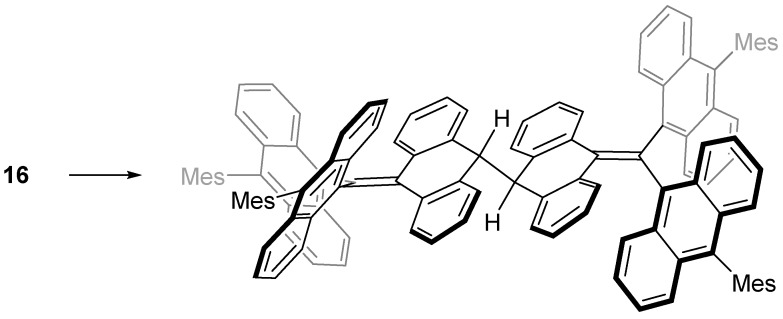
Dimerization reaction of **16**. Mes = mesityl.

**Figure 16:**
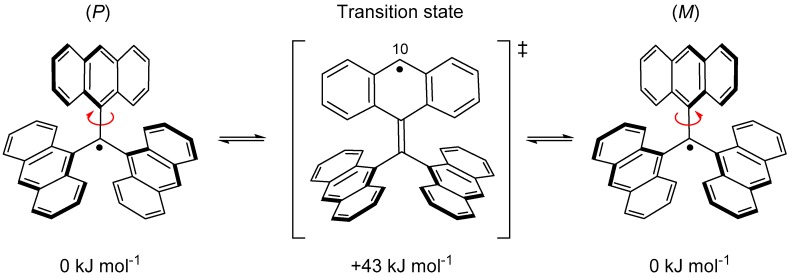
Relative energies for propeller flipping motion of **14** calculated at the UωB97X-D/6-31G** level of theory.

However, the radical that could be isolated in the solid state was not **14** but **15**, where the carbon atom at the 10-position of all anthryl groups was sterically protected by a mesityl group. The stability of **15** was extraordinarily high and showed no decomposition even in air at room temperature. The absence of attack of molecular oxygen against the central carbon indicates that the steric protection by the anthryl groups works very effectively. However, it was found that **16**, which removed one of the three mesityl groups, immediately undergoes a dimerization reaction at the 10-position of the non-substituted anthryl group (Figure 15). According to a UωB97X-D/6-31G** calculation, the flipping motion of the anthryl blade in **14** occurs very rapidly because of the small Δ*H*^‡^ (43 kJ mol^−1^), as shown in Figure 16. Near the transition state, an unpaired electron is mostly localized at the 10-position of the anthryl group, and this localized radical species shows high reactivity with the σ-dimer.

## 5. Conclusions

Our recent studies on stable, π-conjugated, carbon-centered, radical species were described. The π-conjugated radical species with high thermodynamic stability were found to be able to show various association modes due to steric and electronic effects of substituent groups. On the other hand, it was found that the anthryl group used for kinetic stabilization has not only a simple, steric, protective effect but also a role as a reaction site accompanied by a large structural change. By utilizing electronic and steric effects of substituent groups, organic radicals could offer a further possibility for developing new reactions [36] and new functionalities for optoelectronics [37], electronics [38], and spintronics [13,16] devices.

## Figures and Tables

**Table 1:** Radical stabilization energies (RSEs) of selected carbon-centered radicals.

Radical	RSE/kJ mol^−1^
Methyl (^•^CH_3_)	0 (as standard)
*tert*-Butyl	−28.3
Allyl	−77.5
Benzyl	−50.4
Cyclopentadienyl	−98.3
Cycloheptatrienyl	−134.1
Triphenylmethyl (**1**)	−103.4
Phenalenyl (**2**)	−201.6
9-Fluorenyl (**7**)	−90.7

**Table 2:** Redox potentials (V vs Fc/Fc^+^) of **11**, **12**, and **13**.

Compounds	*E*_ox_/V	*E*_red_/V
**11**	+0.28	−1.11
**12**	−0.10	−1.76
**13**	−0.30	−1.78
